# Spatial Representativeness of Environmental DNA Metabarcoding Signal for Fish Biodiversity Assessment in a Natural Freshwater System

**DOI:** 10.1371/journal.pone.0157366

**Published:** 2016-06-30

**Authors:** Raphaël Civade, Tony Dejean, Alice Valentini, Nicolas Roset, Jean-Claude Raymond, Aurélie Bonin, Pierre Taberlet, Didier Pont

**Affiliations:** 1 Hydrosystems and Bioprocesses Research unit, IRSTEA, Antony, France; 2 SPYGEN, Le Bourget du Lac, France; 3 Rhône-Alpes Regional Direction, ONEMA, Bron, France; 4 Laboratoire d'Ecologie Alpine (LECA), CNRS, Grenoble, France; 5 Laboratoire d'Ecologie Alpine (LECA), Université Grenoble-Alpes, Grenoble, France; Swansea University, UNITED KINGDOM

## Abstract

In the last few years, the study of environmental DNA (eDNA) has drawn attention for many reasons, including its advantages for monitoring and conservation purposes. So far, in aquatic environments, most of eDNA research has focused on the detection of single species using species-specific markers. Recently, species inventories based on the analysis of a single generalist marker targeting a larger taxonomic group (eDNA metabarcoding) have proven useful for bony fish and amphibian biodiversity surveys. This approach involves *in situ* filtering of large volumes of water followed by amplification and sequencing of a short discriminative fragment from the 12S rDNA mitochondrial gene. In this study, we went one step further by investigating the spatial representativeness (i.e. ecological reliability and signal variability in space) of eDNA metabarcoding for large-scale fish biodiversity assessment in a freshwater system including lentic and lotic environments. We tested the ability of this approach to characterize large-scale organization of fish communities along a longitudinal gradient, from a lake to the outflowing river. First, our results confirm that eDNA metabarcoding is more efficient than a single traditional sampling campaign to detect species presence, especially in rivers. Second, the species list obtained using this approach is comparable to the one obtained when cumulating all traditional sampling sessions since 1995 and 1988 for the lake and the river, respectively. In conclusion, eDNA metabarcoding gives a faithful description of local fish biodiversity in the study system, more specifically within a range of a few kilometers along the river in our study conditions, i.e. longer than a traditional fish sampling site.

## Introduction

Environmental DNA (eDNA) corresponds to the DNA extracted from an environmental sample such as soil, water or feces without first isolating any target organisms. Total eDNA contains cellular DNA originating from living cells or organisms, and extracellular DNA resulting from natural cell death and subsequent destruction of cell structure [1]. In the last few years, eDNA analysis has drawn the attention of many ecologists as it is non-invasive [2,3], cost-effective [4,5], more sensitive than traditional methods [6], and useful for monitoring and conservation purposes [3,6–10]. Environmental DNA can be used either to detect single invasive or endangered species with species-specific markers, or to describe species diversity for a given taxonomic group using a generalist molecular marker (eDNA metabarcoding [1]).

In freshwater environments, the eDNA metabarcoding approach is increasingly adopted both in mesocosm and *in situ* experiments [5,11–14], and it is particularly interesting for large scale biomonitoring as recommended by recent European directives (*e*.*g*. Water Framework Directive, 2000 [15]). Recently, Valentini *et al*. [13] proposed a new eDNA metabarcoding workflow based on markers targeting fish and amphibians, which were validated *in silico* and *in vitro*. Compared with traditional *in situ* sampling, eDNA analysis was found to be more efficient to assess species richness. However, in addition to the issues inherent to any DNA study such as contaminations or PCR errors, eDNA studies suffer from specific limitations that should be acknowledged and properly assessed [16–18]. This is necessary to ensure reliability of the results and, ultimately, to interpret the eDNA signal detected in natural aquatic ecosystems [16–18].

In mesocosms, eDNA has been shown to remain detectable from a few days to a few weeks after its release in water [19–22]. Nonetheless, mesocosm studies remain imperfect, as it is difficult to model the complexity of natural ecosystems with all the factors potentially involved [16]. For example, eDNA of target species is usually in higher concentrations in mesocosms than in natural ecosystems. In the case of river ecosystems, the distance of detection depends not only on eDNA persistence, but also on the water flow and can therefore be highly variable. For example, Pilliod *et al*. [23] could not detect eDNA released by giant salamanders further than five meters downstream from the location where these animals were placed. On the other hand, in natural lake communities of bivalves and zooplankton, Deiner and Altermatt [24] detected eDNA up to ten kilometers downstream from the lake outlet. There is therefore a need for further research on the persistence and distance of eDNA signal detection in freshwater systems. From an ecological point of view, it is of primary importance to determine the spatial representativeness of the eDNA signal, in particular for fish communities because of the capability of the water flow to disperse eDNA downstream within the river network [25]. Is this signal representative of local communities found in a given river reach, or does it describe species richness more generally at the catchment or sub-catchment scale?

In this paper, we aim to test the potential of eDNA metabarcoding signal for describing the spatial organization of fish communities along a longitudinal gradient, from a lake to the outflowing river, using the workflow proposed by Valentini *et al*. [13]. First, results from simultaneous samplings using both eDNA metabarcoding and traditional “quantitative” methods were compared for each sampling site. Second, we compared the eDNA metabarcoding signals recovered from the lake and from the river with the cumulated list of species detected within each ecosystem during the last 16 years with traditional methods, with the ultimate aim to obtain the most exhaustive list of species detected within the study sites over time. Finally, the distance of eDNA detection and its capacity to describe the structure of fish biodiversity at the water body and catchment scales were discussed.

## Materials and Methods

### Study area and sampling sites

**Fig 1:**
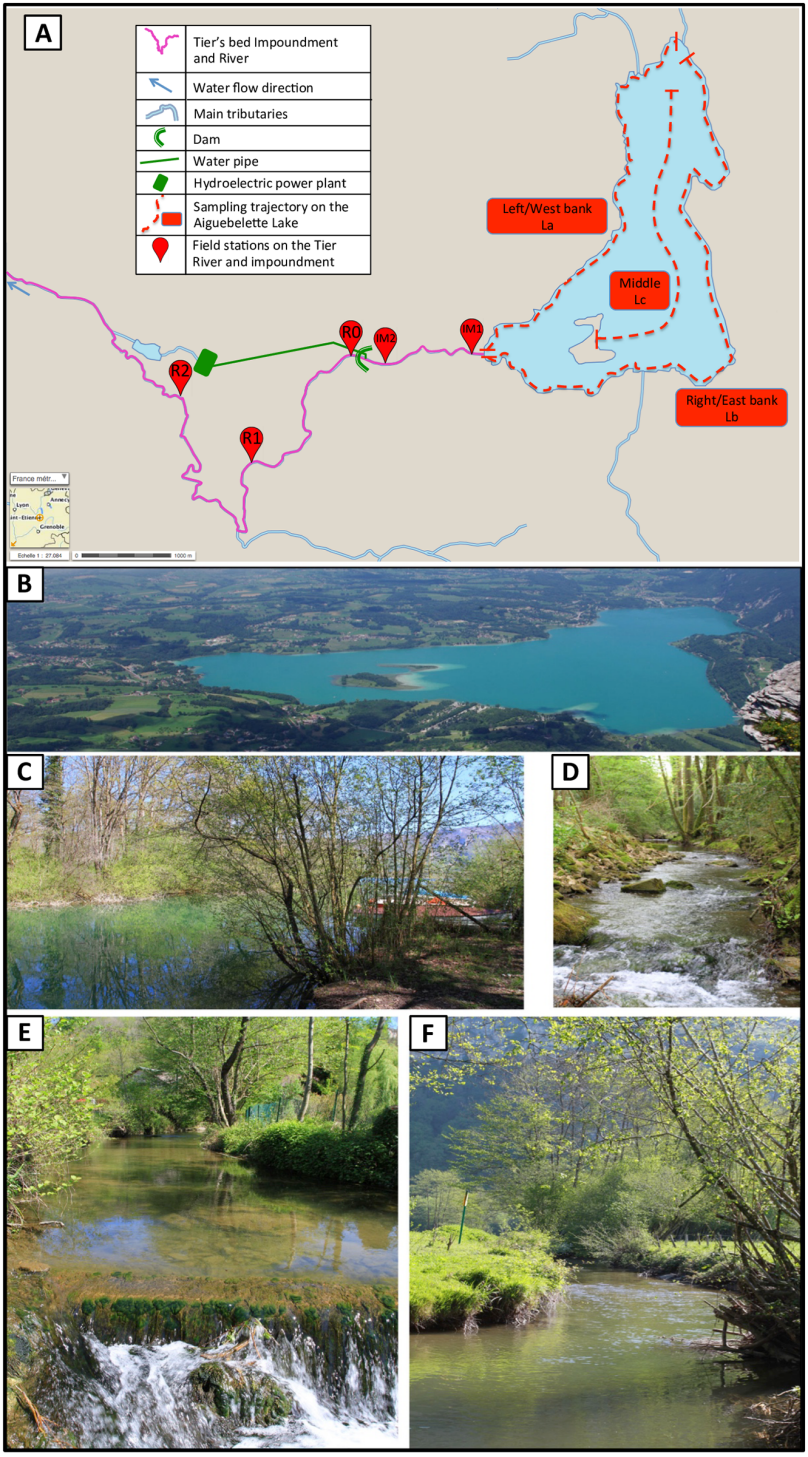
Studied catchment area and localization of the sampling sites. (A) Map of the studied catchment area and localization of the sampling sites: The dotted red lines show the eDNA sampling trajectory on the Aiguebelette lake: La = Left/West bank (4.7 km) trajectory, Lb = Right/East bank (6,7 km) trajectory, Lc = Middle (3,5 km) trajectory; IM1 and IM2, sampling sites on the impoundment respectively 0.2 km and 1.3 km from the lake outlet; R0, R1 and R2, sampling sites on the Tier river respectively 1.7, 3.6 and 6.9 km downstream from the lake outlet. Pictures of Aiguebelette Lake from the south side (B), impoundment at IM1 (C) and Tier River at R0 (D), R1 (E) and R2 (F). Photo credits: M. Bouron (B) and R. Civade (C to F).

Sampling was performed along a catchment basin, from a lake (Aiguebelette) to a unique outflowing river (Tier). Lake Aiguebelette (e.g., Fig 1) is a mono- to dimictic natural lake located 80 km east from Lyon (France), in a piedmont situation between the Jura and Alps mountains. It belongs to the upper Rhône river basin, is located about 375 m above sea level, and has a surface area of 545 ha, an upstream drainage area of 42 km^2^, a maximum depth of 71 m (mean 30 m) and a turnover time of three years [26]. It is a Natura 2000 protected area (Habitat Directive; FR8201770). Fish community is diversified (around 20 species) and dominated by whitefish (*Coregonus lavaretus*), roach (*Rutilus rutilus*) and perch (*Perca fluviatilis*) [27].

The Tier River is the unique outlet of this lake (e.g., Fig 1). A dam situated 1.6 km downstream from the outlet delimits an impoundment. Each night, the water of the Tier impoundment is diverted through a pipe (at 8 m^3^.s^-1^ during 2.5 hours) towards a hydroelectric power plant located 8 km downstream. All the water contained in the Tier impoundment is renewed daily and the water flowing through the dam (residual flow) is 0.171 m^3^.s^-1^ [27]. Downstream from the dam, the first seven kilometers of the Tier River are characterized by a regulated and residual flow (low water depth, river width less than 10 m and a slow flowing reach succeeding to a fast flowing reach; Table 1). Coarse sediments (rocks and boulders) dominate the fast flowing reach whereas the downstream reach has more diversified substrates (cobbles, pebbles, gravel, and fine sediments). From upstream to downstream, the fish community shifts from a “cyprinid” fish community with roach, perch, pike (*Esox lucius*) and dace (*Leuciscus leuciscus*), to a more “salmonid” one with brown trout (*Salmo trutta fario*) and gudgeon (*Gobio gobio*).

**Table 1:** Sampling site characteristics for the Aiguebelette lake, the impoundment and the Tier River downstream from the dam.

Hydrosystem part	Aiguebellette Lake	Impoundment		Tier River		
Field station	Lake	IM1	IM2	R0	R1	R2
**Distance from lake outlet (km)**	**-**	**0.2**	**1.3**	**1.7**	**3.6**	**6.9**
**Traditional sampling**	**n = 1** * **(29/09 to 03/10/2014)**	**-**	**-**	**-**	**n = 1** ** **(30/04/2014)**	**n = 1** ** **(13/05/2014)**
**eDNA sampling**	**n = 3 (29/09/2014)**	**n = 1 (29/09/2014)**	**n = 1 (29/09/2014)**	**n = 3 (25/04/2014)**	**n = 6 (28/04/2014)**	**n = 6 (12/05/2014)**
**Latitude (WGS94)**	**45°33’09.2”N**	**45°32'37.5N**	**45°32'33.6"N**	**45°32'34.9"N**	**45°31’58.3” N**	**45°31’51.4” N**
**Longitude (WGS94)**	**5°47’57.5”E**	**5°46'26.7"E**	**5°45'40.4"E**	**5°45'18.2"E**	**5°44’35.1”E**	**5°44’05.3”E**
**Altitude (m)**	**375**	**375**	**375**	**370**	**300**	**265**
**Slope (m.km-1)**	**-**	**3**	**3**	**30**	**33**	**4**
**Sampled length (m)**	**-**	**-**	**-**	**54**	**120**	**150**
**Width (m)**	**-**	**18**	**18**	**2**	**6.3**	**6.7**
**Depth (m)**	**mean = 30 max = 70**	**1.8**	**1.8**	**0.2**	**0.30**	**0.32**
**Discharge (m3.s-1)**	**-**	**0.171–8.00**	**0.171–8.00**	**0.171**	**0.217**	**0.441**

Sampling effort *n*, number of eDNA filtrations per number of traditional sampling campaigns (using electrofishing in the river and gillnets in the lake).

*survey sampling.

**complete sampling.

The pelagic (Lc) and littoral (La and Lb) zones of the lake were sampled using both the traditional (multimesh gillnets) and eDNA methods. Along the Tier River, eDNA sampling and complete electrofishing were performed at sampling sites selected in the different hydromorphological reaches: R1 (3.6 km downstream from the lake outlet) with the presence of highly turbulent flows and rocks emerging from the water surface (lotic habitat); and R2 (6.9 km downstream from the lake outlet) with smooth water and occasional sand banks (lentic habitat). In addition, the impoundment (IM1 and IM2 sites) and the river 100 m away from the dam outflow (R0) were sampled using the eDNA method only, in order to estimate the persistence of eDNA signal in the vicinity of the lake outlet.

### Ethic statement

The authorization required by the Article L436-9 of Environment Code for the exceptional capture of fish for scientific or ecological perspectives has been previously asked and delivered to the ONEMA technical teams (The French National Agency for Water and Aquatic Environments) by local administration authorities (Departmental Direction of Territories). More precisely, every traditional sampling (gillnet, electrofishing) was carried out in full accordance with the French Environment Policy (including water policy), European standards (EN14011, EN14757) and specific articles dealing with freshwater angling, which particularly aim to protect fish fauna. Moreover, the ONEMA teams have been for years responsible of fish monitoring at a national scale. They have excellent skills in freshwater fish sampling, are educated in animal experiment and are particularly aware of fish well-being. For each sampling site, authorizations were obtained from private landowners and owners of fishing rights (if different) before sampling. Due to the presence of a hydroelectric power plant, a specific permit was granted by Electricité de France (EDF, also partner of the study).

### Traditional fish sampling

The two sampling sites R1 and R2 are conformed to European standards for fish sampling [28], i.e. a river length (150 m) equal to 20-fold the river width, to encompass the different hydraulic habitat units (riffle, pool, etc.). A population sampling depletion method was performed at R1 (30/04/2014) and R2 (13/05/2014) for assessing the absolute species richness at each of the two wadable sites [29]. When no natural barrier existed (R2), stop nets were placed at the upstream and downstream limits of the study site to prevent fish escape during sampling. Using an electrofishing equipment (Heron electrofisher device from Dream Electronics producing a 290 to 400 V and 1.2–1.3 kWa rectified DC current), a three-pass depletion sampling was then operated using two anodes (one anode per 4–5 meter width) and three hand nets to collect all stunned fish. The removed fish were kept alive and finally released in the river after being measured, weighed and identified to the species level [26].

Within the lake, fish were sampled from 29/09/2014 to 03/10/2014 by multimesh gillnets (58 benthic gillnets with 5–55 mm multi-mesh sizes and 22 pelagic gillnets with 6.25–55 mm multi-mesh sizes) following the European standard EN14757 [30]. A random sampling was performed within each depth stratum on both the pelagic and the littoral zones in order to consider the spatial stratification of the water body.

To obtain a list of fish species as comprehensive as possible for both ecosystems (Aiguebelette lake and Tier river), inventories from all available previous sampling sessions were gathered in addition to the sampling sessions realized in 2014: six sessions using multimesh gillnets on the lake (1995, 1996, 2005, 2006, 2007 and 2009), 11 sessions using electrofishing at sites R0 (2015), R1 (2001), R2 (1988 and 2003), and 4.6 km (1997, 2015), 5.4 km (1988, 1997, 2003) and 8.8 km (1988, 1996) from the lake outlet [27,31,32] (http://www.image.eaufrance.fr/poisson/cours/p-ce-resultats.htm). This is hereafter referred to as “cumulated traditional sampling”.

### eDNA metabarcoding analysis

Each eDNA sampling was performed using a filtration device composed of a peristaltic pump (nominal flow of 1.67 L.min^-1^), a filtration capsule (Envirochek HV 1 μm, Pall Corporation, Ann Arbor, MI, USA) and disposable sterile tubing for each sample following the protocol proposed by Valentini *et al*. [13]. Each filtration was timed at 36 min for a water volume of approximately 45 L.

In order to avoid potential contaminations due to traditional survey equipment and to perform traditional and eDNA samplings in the narrowest possible time window, eDNA sampling was implemented just before gillnets survey on the Aiguebelette Lake and less than 48 h before electrofishing in the Tier River sampling sites (R1 and R2; Table 1).

The sampling effort varied according to sampling sites in order to better consider the levels of heterogeneity of the different environments studied (Table 1). A relatively large sampling effort was implemented for comparison sites (i.e. sites for which simultaneous eDNA and traditional samplings were performed): three samples for the lake (La, Lb, Lc) and six samples for each of the comparison river sites (R1a to R1f and R2a to R2f). A lower sampling effort was implemented for control sites (i.e. sites selected to estimate the persistence of eDNA signal in the vicinity of the lake outlet): one sample per impoundment site (IM1, IM2) and three samples for the river site 100 m away from the dam outflow (R0a to R0c).

On the Aiguebelette Lake, three filtrations were performed (29/09/2014) along trajectories in the following order: the first one from the northern part of the lake to the impoundment following the western bank at a distance of 20 m (La), the second one from the impoundment to its northern part following the eastern bank at a distance of 20 m (Lb) and the third one from the northern part of the lake to the two islands passing to the lake’s deeper points and avoiding banks (Lc). For these filtrations, the boat’s speed was adapted to the trajectory to maintain a filtration time of 36 min. Each filtration was performed from the boat’s bow in order to avoid potential contaminations. The sampling session was organized so as to avoid perturbations due to the boat movement.

On the Tier Impoundment (IM1 and IM2), one filtration was performed at the middle of each sampling site (29/09/2014). On the Tier River, at sampling site R0, three filtrations were performed (25/04/2014). At sampling sites R1 (28/04/2014) and R2 (12/05/2015), six filtrations were performed per sampling site. All filtrations were performed upstream from the operator to avoid contaminations. No field controls were used during this experiment but with the same sampling protocol, Valentini *et al*. (2016) [13] did not detect any false positives in fishless lakes.

At the end of each filtration, the water inside the capsule was emptied and the capsule was filled with a preservation buffer (Tris–HCl 0.1 M, EDTA 0.1 M, NaCl 0.01 M and N-lauroyl sarcosine 1% with pH 7.5–8), stored in a sterile and disposable bag at 4°C in the dark until the end of the day and finally stored at room temperature before DNA extraction. For DNA extraction, filtration capsules were left at 56°C for 2 h, agitated manually for 5 min and then emptied into three 50 mL tubes. In total, approximately 120 mL were retrieved in three tubes that were centrifuged for 15 min at 15,000 g. Supernatant was removed with a sterile pipette, leaving 15 mL of liquid at the bottom of the tube. Subsequently, 33 mL of ethanol and 1.5 mL of 3M sodium acetate were added to each 50 mL tube. The three tubes were centrifuged at 15 000 g for 15 min at 6°C and the supernatant was discarded. After this step, 360 μL of ATL Buffer of the DNeasy Blood & Tissue Extraction Kit (Qiagen) were added to the first tube, the tube was vortexed and the supernatant was transferred to the second tube [33]. This operation was repeated for all tubes. The supernatant of the third tube was finally transferred to a 2 mL tube and the DNA extraction was performed following the manufacturer’s instructions. Three negative extraction controls were also performed. They were amplified and sequenced in the same way and in parallel to the samples to monitor possible contaminations.

DNA amplifications were performed in a final volume of 25 μL, using 3 μL of DNA extract as template. The amplification mixture contained 1 U of AmpliTaq Gold DNA Polymerase (Applied Biosystems, Foster City, CA), 10 mM of Tris-HCl, 50 mM of KCl, 2.5 mM of MgCl2, 0.2 mM of each dNTP, 0.2 μM of “teleo” primers [13], 4 μM of human blocking primer for “teleo” primers [13] and 0.2 μg/μL of bovine serum albumin (BSA, Roche Diagnostic, Basel, Switzerland). The “teleo” primers were 5’-labeled with a seven-nucleotide tag unique to each sample (with at least three differences between any pair of tags) allowing the assignment of each sequence to the corresponding sample during sequence analysis. Tags for forward and reverse primers were identical for each sample. The PCR mixture was denatured at 95°C for 10 min, followed by 50 cycles of 30 s at 95°C, 30 s at 55°C and 1 min at 72°C, and followed by a final elongation at 72°C for 7 min, in a room dedicated to amplified DNA, with negative air pressure and physically separated from the DNA extraction rooms. Twelve replicate PCRs were run per sample. Three negative PCR controls (ultrapure water, with 12 replicates as well) were analyzed in parallel to the samples to monitor possible contaminations during the PCR step.

After amplification, samples were titrated using capillary electrophoresis (QIAxcel; Qiagen GmbH, Hilden, Germany) and purified using a MinElute PCR purification kit (Qiagen GmbH, Hilden, Germany). Before sequencing, purified DNA was titrated again using capillary electrophoresis. The purified PCR products were pooled in equal volumes, to achieve an expected sequencing depth of 300,000 reads per sample. Library preparation and sequencing were performed at Fasteris (Geneva, Switzerland; https://www.fasteris.com/dna/). Libraries were prepared using the TruSeq Nano DNA genomic kit (Illumina, San Diego, CA, USA) and a paired-end sequencing (2x100 bp) was carried out using an Illumina MiSeq sequencer (Illumina, San Diego, CA, USA) using the Paired-end MiSeq Reagent Kit V2 (Illumina, San Diego, CA, USA) following the manufacturer’s instructions. In total, two MiSeq runs were performed.

Sequence reads were analyzed using the programs implemented in the OBITools package (http://metabarcoding.org/obitools; [34]) as described in De Barba *et al*. (2014) [35]. The program *illuminapairedend* was used to assemble the forward and reverse reads corresponding to a single amplicon. No special threshold was applied after the alignment step, the bad alignments being removed implicitly during the following filtering steps. Subsequently, the *ngsfilter* program identified primers and tags and assigned the sequences to each sample. This program was used with its default parameters tolerating two mismatches for each of the two primers and no mismatch for the tags. A separate dataset was created for each sample by splitting the original dataset in several files using *obisplit*. After this step, each sample was analyzed individually up to the ecological analyses. Strictly identical sequences were clustered together using *obiuniq*. Sequences shorter than 20 bp, or occurring less than 10 times were excluded using the *obigrep* program. The *obiclean* program was then run to assign the status of “head”, “internal” or “singleton” to each sequence, within a PCR product. All sequences labeled “internal”, corresponding most likely to PCR errors were discarded. The taxonomic assignment of MOTUs was performed using the program *ecotag*, with both the local reference database of Teleostei built in [13] and the sequences extracted from the release 118 (standard sequences) of the EMBL database using the *ecoPCR* program [36,37]. MOTUs showing less than 98% similarity with either the local or the EMBL reference databases were removed. Taxa were preferentially assigned based on the local reference database, except if the similarity was higher for the EMBL reference database. Finally, to take into account bad assignation of a few numbers of sequences to the wrong sample [38], all sequences with a frequency of occurrence below 0.003 per taxon and per sequencing run for fish were discarded. These thresholds were empirically determined to clear all reads from the negative controls included in our global data production procedure [35].

For the negatives controls, 240,737 reads were obtained: 2,756 for the extraction negative controls and 237,981 for PCR negative controls. After filtering, all extraction and PCR negative controls analysed were empty of reads.

### Comparison of simultaneous sampling with eDNA and traditional methods in 2014

Within the Tier River reaches, the fish community was simultaneously assessed by six eDNA metabarcoding samples and with a complete electrofishing multi-pass removal approach (i.e. higher sampling effort than classical electrofishing) at two sampling sites (R1 and R2). The species lists obtained by each method were compared on a presence-absence basis. A similar comparison between the results of the two approaches was also performed for the lake.

### eDNA metabarcoding vs cumulated traditional sampling for species detection

Two lists of species detected by eDNA metabarcoding in 2014, the first one cumulating the detections of eDNA samples for the comparison site on the lake (La, Lb, Lc) and the second one cumulating the detections of eDNA samples at the comparison sites R1 and R2 on the river (R1a to R1f, R2a to R2f), were respectively compared to the cumulated traditional sampling list for the lake (1995 to 2014) and the other one for the river (1988 to 2015).

### Statistical analyses

All statistical analyses were performed with the R program (version 3.1.3). The molecular marker used for fish detection does not discriminate between *Telestes souffia*, *Chondrostoma nasus* and *Chondrostoma toxostoma*, so these three species are referred to as Species Group 1 (SG1).

A correspondence analysis (CA) was performed on species presence-absence data to analyze the spatial distribution of the eDNA metabarcoding signal along the lake-river gradient. To avoid bias in the analysis, SG1 detections were not taken into account for the CA, because it represents one detection for eDNA, but several species in the traditional methods’ list. First, only eDNA metabarcoding data were used to compute the CA using the ade4 R library version 1.7–2 [39]. Then, adding all 2014 traditional sampling sessions, the compilation of all species occurrences since 1988 and each of these previous sampling sessions separately allowed comparing the patterns of fish species spatial distribution obtained by eDNA metabarcoding *versus* traditional approaches.

## Results

### Comparison of simultaneous sampling with eDNA and traditional methods in 2014

In the lake, a total of 22 taxa were detected, among which 12 were revealed by both methods (Table 2). Of these 12 taxa, 10 were found in all eDNA metabarcoding samples. Nine taxa were identified only by eDNA metabarcoding and one only by the multimesh gillnets method.

**Table 2:** Fish species detected using eDNA metabarcoding (three filtrations) and gillnets during the sampling campaigns operated together in 2014 in the Aiguebelette lake.

Fish taxa		eDNA metabarcoding			Gillnets sampling
Latin name	Common name		Number of reads		Detections
		La	Lb	Lc	
***SHARED TAXA (n = 12)***					
***Perca fluviatilis***	**Perch**	**162580**	**79487**	**91657**	**X**
***Coregonus lavaretus***	**Lavaret**	**125075**	**30264**	**11430**	**X**
***Rutilus rutilus***	**Roach**	**61138**	**46706**	**18619**	**X**
***Esox lucius***	**Pike**	**23428**	**30686**	**15205**	**X**
***Scardinius erythrophthalmus***	**Rudd**	**35596**	**10092**	**16013**	**X**
***Squalius cephalus***	**Chub**	**19566**	**11728**	**8608**	**X**
***Abramis brama***	**Bream**	**5129**	**4286**	**4779**	**X**
***Tinca tinca***	**Tench**	**4248**	**1290**	**2534**	**X**
***Lepomis gibbosus***	**Pumpkinseed**	**5101**	**1048**	**224**	**X**
***Cyprinus carpio***	**Carp**	**803**	**2149**	**3033**	**X**
***Gobio spp.***	**-**	**1951**	**2992**		**X**
***Salvelinus spp.***	**-**	**5904**			**X**
***TAXA DETECTED USING eDNA ONLY (n = 9)***					
***Leuciscus spp.***	**-**	**16637**	**13139**	**167**	
***Alburnus alburnus***	**Bleak**	**8189**	**859**	**2702**	
***Salaria fluviatilis***	**Freshwater blenny**	**3678**	**3992**	**255**	
***Salmo trutta***	**Trout**	**19**		**912**	
***Micropterus sp.***	**-**	**665**			
***Carassius spp.***	**-**	**510**			
***Cottus spp.***	**-**	**23**		**14**	
***Barbus barbus***	**Barbel**	**12**			
***Barbatula sp.***	**-**	**11**			
***SPECIES CAUGHT BY TRADITIONAL SAMPLING ONLY (n = 1)***					
***Sander lucioperca***	**Pikeperch**				**X**
**Total richness**			**21**		**13**

#### Number of reads per species and per filtration

At the R1 site on the Tier River, 16 taxa were uncovered in total, among which 10 were identified by both methods (Table 3). Among these shared detections, eight were identified in all six eDNA metabarcoding samples. Six taxa were detected only by eDNA metabarcoding. At the R2 sampling site, a total of 19 taxa were observed, with 14 taxa identified by both methods (Table 4). Among these 14 taxa, 13 were detected in all six eDNA metabarcoding samples. Five taxa were specific to eDNA metabarcoding.

**Table 3:** Fish species detected using eDNA metabarcoding (six filtrations) and complete electrofishing during the sampling campaigns operated together in 2014 at the R1 site.

**Fish taxa**		**eDNA metabarcoding approach**						**electrofishing sampling**
**Latin name**	**Common name**			**Number of reads**				**Detections**
		**R1a**	**R1b**	**R1c**	**R1d**	**R1e**	**R1f**	
***SHARED TAXA (n = 10)***								
***Rutilus rutilus***	**Roach**	**30883**	**72062**	**38571**	**52227**	**22037**	**95338**	**X**
***Salmo trutta***	**Trout**	**26227**	**22944**	**29873**	**56015**	**15028**	**57358**	**X**
***Barbatula sp*.**	**-**	**2569**	**3882**	**5235**	**4813**	**1801**	**9748**	**X**
***Teletes souffia***	**Riffle dace**	3780*	3468*	2512*	8532*	1909*	4501*	**X**
***Squalius cephalus***	**Chub**	**823**	**705**	**814**	**2155**	**535**	**1634**	**X**
***Phoxinus phoxinus***	**Minnow**	**42**	**60**	**134**	**83**	**63**	**286**	**X**
***Perca fluviatilis***	**Perch**	**52**	**84**	**177**	**176**	**29**	**20**	**X**
***Lepomis gibbosus***	**Pumpkinseed**	**56**	**54**	**77**	**230**	**13**	**47**	**X**
***Alburnoides bipunctatus***	**Spirlin**				**276**			**X**
***Gobio spp*.**	**-**			**14**	**53**		**64**	**X**
***TAXA DETECTED USING eDNA ONLY (n = 6)***								
***Barbus barbus***	**Barbel**		**61**		**455**			
***Scardinius erythrophthalmus***	**Rudd**						**33**	
***Cottus spp*.**	**-**				**19**			
***Alburnus alburnus***	**Bleak**					**17**		
***Oncorhynchus mykiss***	**Rainbow trout**	**14**						
***Leuciscus spp*.**	**-**			**12**				
**Total richness**				**16**				**10**
**Fish taxa**		**eDNA metabarcoding approach**						**electrofishing sampling**
**Latin name**	**Common name**			**Number of reads**				**Detections**
		**R1a**	**R1b**	**R1c**	**R1d**	**R1e**	**R1f**	
***SHARED TAXA (n = 10)***								
***Rutilus rutilus***	**Roach**	**30883**	**72062**	**38571**	**52227**	**22037**	**95338**	**X**
***Salmo trutta***	**Trout**	**26227**	**22944**	**29873**	**56015**	**15028**	**57358**	**X**
***Barbatula sp*.**	**-**	**2569**	**3882**	**5235**	**4813**	**1801**	**9748**	**X**
***Teletes souffia***	**Riffle dace**	3780*	3468*	2512*	8532*	1909*	4501*	**X**
***Squalius cephalus***	**Chub**	**823**	**705**	**814**	**2155**	**535**	**1634**	**X**
***Phoxinus phoxinus***	**Minnow**	**42**	**60**	**134**	**83**	**63**	**286**	**X**
***Perca fluviatilis***	**Perch**	**52**	**84**	**177**	**176**	**29**	**20**	**X**
***Lepomis gibbosus***	**Pumpkinseed**	**56**	**54**	**77**	**230**	**13**	**47**	**X**
***Alburnoides bipunctatus***	**Spirlin**				**276**			**X**
***Gobio spp*.**	**-**			**14**	**53**		**64**	**X**
***TAXA DETECTED USING eDNA ONLY (n = 6)***								
***Barbus barbus***	**Barbel**		**61**		**455**			
***Scardinius erythrophthalmus***	**Rudd**						**33**	
***Cottus spp*.**	**-**				**19**			
***Alburnus alburnus***	**Bleak**					**17**		
***Oncorhynchus mykiss***	**Rainbow trout**	**14**						
***Leuciscus spp*.**	**-**			**12**				
**Total richness**				**16**				**10**

Number of reads per species and per filtration.

*The molecular marker does not discriminate between *Telestes souffia*, *Chondrostoma nasus* and *Chondrostoma toxostoma*.

**Table 4:** Fish species detected using eDNA metabarcoding (six filtrations) and complete electrofishing during the sampling campaigns operated together in 2014 at the R2 site.

Fish taxa		eDNA metabarcoding approach (n = 6)						Simultaneous sampling
Latin name	Common name			Number of reads				Detections
		R2a	R2b	R2c	R2d	R2e	R2f	
***SHARED TAXA (n = 14)***								
***Telests souffia***	**Riffle dace**	5492*	6195*	3331*	12527*	5424*	24793*	**X**
***Alburnoides bipunctatus***	**Spirlin**	**1185**	**3890**	**4827**	**7366**	**4990**	**32745**	**X**
***Barbus barbus***	**Barbel**	**3462**	**6765**	**3194**	**8365**	**2133**	**18346**	**X**
***Squalius cephalus***	**Chub**	**1273**	**3067**	**1463**	**2608**	**975**	**6551**	**X**
***Salmo trutta***	**Trout**	**1273**	**2854**	**1405**	**2712**	**978**	**4104**	**X**
***Barbatula sp.***	**-**	**618**	**1487**	**560**	**1126**	**308**	**2486**	**X**
***Gobio spp.***	**-**	**232**	**731**	**259**	**532**	**170**	**1617**	**X**
***Rutilus rutilus***	**Roach**	**174**	**545**	**194**	**840**	**86**	**1112**	**X**
***Oncorhynchus mykiss***	**Rainbow trout**	**395**	**358**	**267**	**388**	**72**	**932**	**X**
***Lampetra spp.***	**-**	**74**	**330**	**132**	**254**	**445**	**759**	**X**
***Phoxinus phoxinus***	**Minnow**	**75**	**207**	**273**	**181**	**37**	**75**	**X**
***Lepomis gibbosus***	**Pumpkinseed**	**112**	**171**	**60**	**107**	**78**	**182**	**X**
***Leuciscus spp.***	**-**	**29**	**43**	**26**	**66**	**13**	**79**	**X**
***Scardinius erythrophthalmus***	**Rudd**		**15**					**X**
***TAXA DETECTED USING eDNA ONLY (n = 5)***								
***Cottus spp.***	**-**	**570**	**941**	**366**	**718**	**273**	**1014**	
***Abramis brama***	**Bream**		**14**				**15**	
***Perca fluviatilis***	**Perch**	**13**					**11**	
***Alburnus alburnus***	**Bleak**						**13**	
***Salaria fluviatilis***	**Freshwater blenny**		**11**					
**Total richness**				**19**				**14**

Number of reads per species and per filtration.

*The molecular marker does not discriminate between *Telestes souffia*, *Chondrostoma nasus* and *Chondrostoma toxostoma*.

### eDNA metabarcoding *vs* cumulated traditional sampling for species detection

For the lake, 26 taxa were detected in total using cumulated traditional sampling and/or eDNA metabarcoding. Of these, 21 were picked up by the three eDNA metabarcoding samples (L1, L2 and L3), against 22 taxa by the seven cumulated traditional sampling campaigns (Table 5). The two methods shared 17 detected taxa. Four taxa were revealed only by eDNA metabarcoding whereas five species were identified only with the traditional method.

**Table 5:** Fish species detected using eDNA metabarcoding (three filtrations in 2014) and the seven gillnets sampling campaigns (from 1995 to 2014) within the Aiguebelette lake.

Fish taxa		eDNA metabarcoding		Gillnets sampling	
Latin name	Common name	Detections	Reads (%)	Detections	Frequency
***SHARED TAXA (n = 17)***					
***Perca fluviatilis***	**Perch**	**X**	37,2821	**X**	F
***Coregonus lavaretus***	**Lavaret**	**X**	18,6306	**X**	F
***Rutilus rutilus***	**Roach**	**X**	14,1278	**X**	F
***Esox lucius***	**Pike**	**X**	7,7440	**X**	F
***Scardinius erythrophthalmus***	**Rudd**	**X**	6,8929	**X**	F
***Squalius cephalus***	**Chub**	**X**	4,4577	**X**	F
***Leuciscus spp.***	**-**	**X**	3,3451	**X**	F
***Abramis brama***	**Bream**	**X**	1,5857	**X**	F
***Alburnus alburnus***	**Bleak**	**X**	1,3127	**X**	C
***Tinca tinca***	**Tench**	**X**	0,9018	**X**	F
***Salaria fluviatilis***	**Freshwater blenny**	**X**	0,8853	**X**	R
***Lepomis gibbosus***	**Pumpkinseed**	**X**	0,7120	**X**	F
***Cyprinus carpio***	**Carp**	**X**	0,6686	**X**	F
***Salvelinus spp.***	**-**	**X**	0,6596	**X**	F
***Gobio spp.***	**-**	**X**	0,5522	**X**	F
***Salmo trutta***	**Trout**	**X**	0,1040	**X**	R
***Barbatula sp.***	**-**	**X**	0,0012	**X**	R
***TAXA DETECTED USING eDNA ONLY (n = 4)***					
***Micropterus sp.***	**-**	**X**	0,0743		
***Carassius spp.***	**-**	**X**	0,0570		
***Cottus spp.***	**-**	**X**	0,0041		
***Barbus barbus***	**Barbel**	**X**	0,0013		
***SPECIES CAUGHT BY TRADITIONAL SAMPLING ONLY (n = 5)***					
***Blicca bjoerkna***	**Silver bream**			**X**	C
***Lota lota***	**Burbot**			**X**	C
***Oncorhynchus mykiss***	**Rainbow trout**			**X**	R
***Sander lucioperca***	**Pikeperch**			**X**	F
***Chondrostoma nasus***	**Nase**			**X**	R
**Total richness**		**21**		**22**	

Relative abundance of reads (in % for a total of 895,133 reads) per species (eDNA metabarcoding) and frequency of species caught with gillnets: frequent species (F), common species (C) and rare species (R) caught in more than 50%, 15–50% and less than 15%, respectively, of the total number of fishing campaigns.

Concerning the Tier River, 21 taxa were detected in total with cumulated traditional sampling and/or eDNA metabarcoding. Of these, 19 and 21 taxa were uncovered by eDNA metabarcoding and electrofishing, respectively (Table 6). All taxa identified within the eDNA metabarcoding samples were also present in the traditional sampling campaigns, and two additional species were recorded with this last method only.

**Table 6:** Fish species detected using eDNA metabarcoding (12 filtrations in 2014) and the 13 electrofishing sampling campaigns (from 1988 to 2015) within the Tier River.

Fish taxa		eDNA metabarcoding		electrofishing sampling	
Latin name	Common name	Detections	Reads (%)	Detections	Frequency
***SHARED TAXA (n = 19)***					
***Rutilus rutilus***	**Roach**	**X**	39,8441	**X**	C
***Salmo trutta***	**Trout**	**X**	28,0080	**X**	F
***Telestes souffia***	**Riffle dace**	**X**	10.4617 *	**X**	F
***Alburnoides bipunctatus***	**Spirlin**	**X**	7,0129	**X**	C
***Barbus barbus***	**Barbel**	**X**	5,4274	**X**	F
***Barbatula sp.***	**-**	**X**	4,3937	**X**	F
***Squalius cephalus***	**Chub**	**X**	2,8675	**X**	F
***Cottus spp.***	**-**	**X**	0,4949	**X**	C
***Gobio spp.***	**-**	**X**	0,4658	**X**	F
***Oncorhynchus mykiss***	**Rainbow trout**	**X**	0,3078	**X**	R
***Lampetra spp.***	**-**	**X**	0,2530	**X**	C
***Phoxinus phoxinus***	**Minnow**	**X**	0,1923	**X**	F
***Lepomis gibbosus***	**Pumpkinseed**	**X**	0,1506	**X**	R
***Perca fluviatilis***	**Perch**	**X**	0,0713	**X**	R
***Leuciscus spp.***	**-**	**X**	0,0340	**X**	R
***Scardinius erythrophthalmus***	**Rudd**	**X**	0,0061	**X**	R
***Alburnus alburnus***	**Bleak**	**X**	0,0038	**X**	R
***Abramis brama***	**Bream**	**X**	0,0037	**X**	R
***Salaria fluviatilis***	**Freshwater blenny**	**X**	0,0014	**X**	R
***SPECIES CAUGHT BY TRADITIONAL SAMPLING ONLY (n = 2)***					
***Esox lucius***	**Pike**			**X**	R
***Thymallus thymallus***	**European grayling**			**X**	R
**Total richness**		**19**		**21**	

Relative abundance of reads (in % of a total of 788,244 reads) per species (eDNA metabarcoding) and frequency of species caught with gillnets: frequent species (F), common species (C) and rare species (R) caught in more than 50%, 15–50% and less than 15%, respectively, of the total number of fishing campaigns.

*The molecular marker does not discriminate between *Telestes souffia*, *Chondrostoma nasus* and *Chondrostoma toxostoma*.

### Longitudinal pattern of fish biodiversity

**Fig 2:**
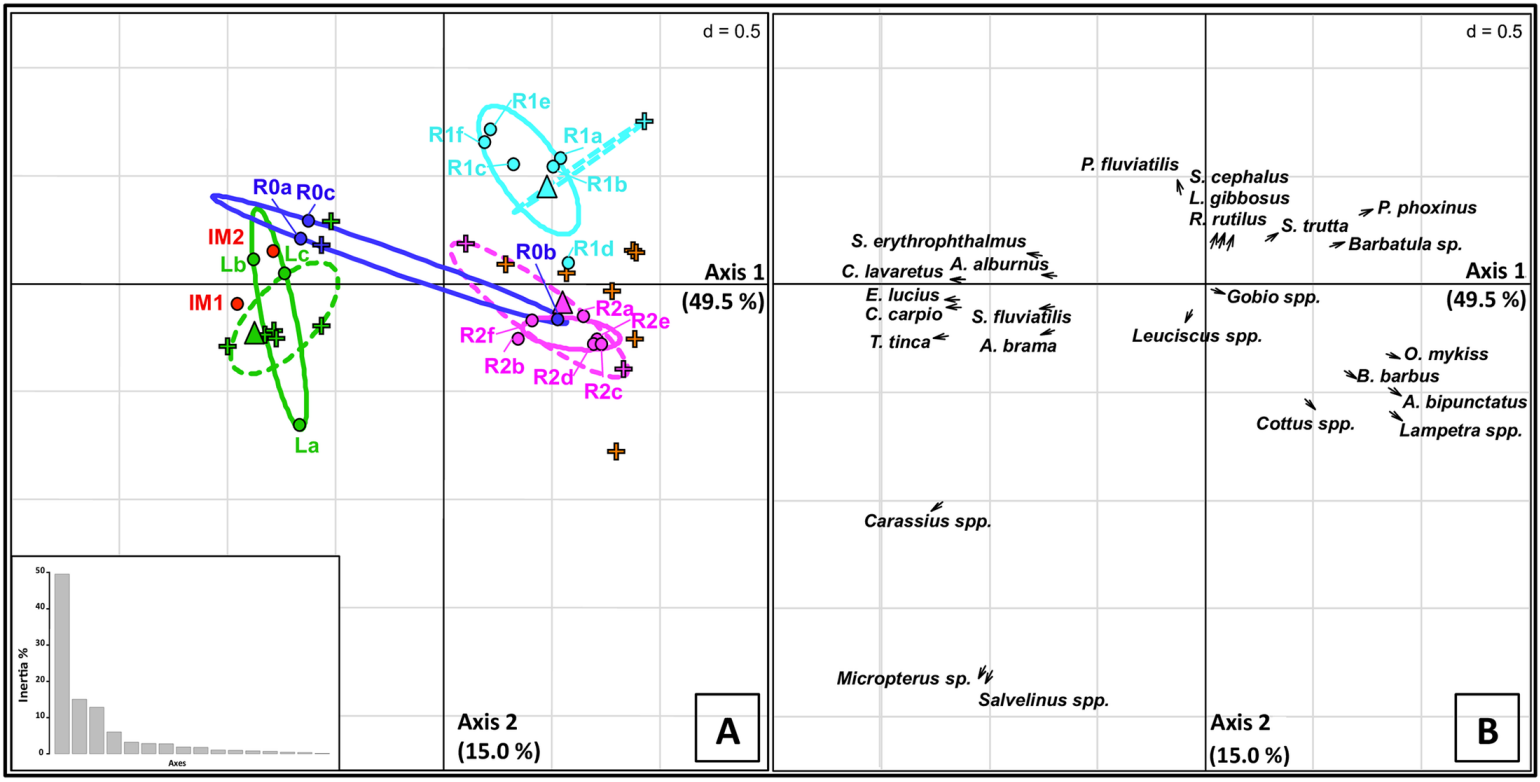
First factorial map of the correspondence analysis based on the whole dataset. Color codes for the different localities sampled in 2014 using eDNA metabarcoding (dots): lake (L, green), impoundment (IM1 and IM2, red), Tier River (R0: dark blue, R1: light blue, R2; pink). The results of the different filtrations are indicated by a letter following the site name: three for L (a-c) and R0 (a-c), six for R1 (a-f) and R2 (a-f). Traditional fish samples added as supplementary individuals (crosses for previous fishing sessions and triangles for simultaneous fishing sessions in 2014): green (Lake), dark blue (R0), light blue (R1), pink (R2) and orange colors (other river sites). The plain and dashed line ellipses define the area encircling 95% of the samples for a given site for the eDNA analysis and the combined traditional fishing campaigns, respectively. Bottom left, histogram of the inertia distribution per axis.

Most of the information contained in the eDNA metabarcoding dataset was accounted by the first two axes of the CA, which represented 49.5% and 15% of the total inertia, respectively (e.g., Fig 2). Environmental DNA sampling sites were split into three groups on the factorial plan. The first axis discriminated between lotic and lentic species and/or between typical river and lake species. The second axis mainly differentiated the two river sites R1 and R2. All samples from the lake and the impoundment were grouped together, whereas samples from R1 and R2 formed two clear extra groups. Interestingly, the R0a and R0c samples from the dam outflow were located close to the impoundment samples and the R0b sample within the R2 sample group. R0a and R0c were characterized by the presence of typical lake species (e.g. *Coregonus lavaretus*), as in the impoundment (Table 7), whereas R0b detected typical riverine rheophilic non salmonid species (*Barbus barbus*, *Phoxinus phoxinus*).

**Table 7:** Fish species detected by eDNA metabarcoding in 2014 within the impoundment (IM1 and IM2 samples) and at the R0 site (downstream from the dam, three filtrations).

Fish taxa		IM—Impoundment		R0—Tier River		
Latin name	Common name	IM1	IM2	R0a	R0b	R0c
***Perca fluviatilis***	**Perch**	**158105**	**97188**	**383**	**106**	**639**
***Rutilus rutilus***	**Roach**	**58107**	**39628**	**23037**	**19855**	**84578**
***Esox lucius***	**Pike**	**30101**	**36902**	**30**		**91**
***Lepomis gibbosus***	**Pumpkinseed**	**16362**	**37991**	**75**	**23**	**217**
***Squalius cephalus***	**Chub**	**15944**	**35406**	**39**	**308**	**113**
***Coregonus lavaretus***	**Lavaret**	**23371**	**328**	**11**		**24**
***Cyprinus carpio***	**Carp**	**7753**	**14064**			**38**
***Tinca tinca***	**Tench**	**4515**	**17184**			
***Scardinius erythrophthalmus***	**Rudd**	**4264**	**11726**	**14**		**27**
***Leuciscus spp.***	**-**	**5738**	**7772**	**126**	**77**	**299**
***Salaria fluviatilis***	**Freshwater blenny**	**3887**	**1027**	**80**	**30**	**230**
***Gobio spp.***	**-**	**4430**	**205**	**11**	**67**	**67**
***Alburnus alburnus***	**Bleak**	**379**	**2218**			
***Abramis brama***	**Bream**	**1134**	**147**	**21**		
***Salmo trutta***	**Trout**		**168**		**142**	**38**
***Carassius spp.***	**-**	**32**				
SG1*	**-**			**60**	**2568**	**218**
***Barbus barbus***	**Barbel**				**1563**	
***Alburnoides bipunctatus***	**Spirlin**				**935**	
***Barbatula sp.***	**-**				**143**	
***Cottus spp.***	**-**				**37**	
***Lampetra spp.***	**-**				**33**	
***Oncorhynchus mykiss***	**Rainbow trout**				**17**	
***Phoxinus phoxinus***	**Minnow**				**15**	
**Total richness**		**16**			**21**	

Number of reads per species and per filtration.

*The molecular marker does not discriminate between *Telestes souffia*, *Chondrostoma nasus* and *Chondrostoma toxostoma*.

The three traditional sampling campaigns achieved in 2014, plotted to the factorial plan as supplementary information, fitted with the eDNA results for both the lake and river communities. A good agreement was observed between the compilation of all species occurrences detected since 1988 using traditional sampling methods and eDNA metabarcoding results describing spatial organization of the lentic-lotic gradient. Furthermore, looking at all traditional sampling campaigns, dispersion among eDNA metabarcoding samples was quite similar to that observed among traditional campaigns (e.g., Fig 2).

## Discussion

In the present study, we investigated the representativeness (i.e. ecological relevance and signal variability in space) and the robustness of the eDNA metabarcoding approach for fish biodiversity assessment, following the method presented by Valentini *et al*. [13]. We demonstrated that this approach is more efficient to detect species presence than a single multimesh gillnets sampling campaign on the lake and also more efficient than a complete electrofishing multi-passes removal in the river. The list of taxa detected by eDNA analysis is comparable to the species list obtained when cumulating all the traditional sampling campaigns since 1995 and 1988 for the lake and the river, respectively. Additionally, it was representative of local fish biodiversity in a range of a few kilometers under our study conditions. The eDNA metabarcoding signal obtained using this workflow is also representative of the longitudinal pattern of fish communities within the studied catchment area, from the lake to the river downstream.

### Ecological relevance of the eDNA metabarcoding signal

In the Tier River and Aiguebelette Lake ecosystems, respectively 91% and 65% of the total number of taxa were identified by both eDNA metabarcoding and cumulated traditional sampling. When focusing on species detected only by eDNA metabarcoding, the situation differs notably between the two ecosystems. In the Tier River, all taxa detected with eDNA metabarcoding are present in the cumulated traditional sampling even if, for six of them, their eDNA signal is very low (*i*.*e*. the percentage of reads is lower than one per thousand). On the contrary, when considering the lake ecosystem, four species are absent from cumulated traditional sampling. Even if they can occasionally be found in lakes, those species are not classified as characteristic of such a large lentic environment [40]. Nevertheless, *Cottus* spp. has already been caught in comparable alpine lakes [41]. In addition, the percentages of reads associated with these species are very low and represent less than one per thousand of the total number of reads. However, a low eDNA metabarcoding signal is not necessarily associated with an absence of detection by cumulated traditional sampling in the lake, as exemplified by *Barbatula* sp. From a methodological perspective, all laboratory quality controls validated this eDNA metabarcoding workflow, confirming the presence of eDNA from these four species in our samples. Moreover, precautions taken during sampling make field contaminations possible but unlikely given the stringent sampling protocol performed. Using field negative controls would be one part of the solution but their reliability remains to be proved [42]. In addition, when the percentage of reads is very low, it is difficult to clearly identify the signal origin: rare species in the sampling site or located upstream, resuspension from sediments, transport by other vectors such as bird excrements [43,44]. One could discuss the need for defining a threshold below which the signal needs to be interpreted with caution. Nevertheless, our example tends to demonstrate that such weak signals could have an ecological significance and further research is needed to better characterize the detection threshold.

The number of species detected only using cumulated traditional sampling sessions is higher in the lake than in the river. Among the five species not detected with eDNA metabarcoding for the lake, three of them are common species (*Blicca bjoerkna*, *Lota lota* and *Sander lucioperca*) and their absence might be due to a limited sampling effort (three filtrations) and the limited spatial covering on the lake, not adapted to the size of this ecosystem. In the case of the two other species (*Chondrostoma nasus*, *Oncorhynchus mykiss*), they were caught only during the 1990s and could have disappeared since. In the river, the two species undetected by eDNA metabarcoding are uncommon (*Thymallus thymallus*, *Esox lucius*). Finally, even if some discrepancies exist between the taxa lists obtained by the two methods, the ecological relevance of eDNA metabarcoding detections are demonstrated. No species having ecological requirements incompatible with either the lake or the river characteristics was detected by eDNA metabarcoding. Nevertheless, our results support the need for increasing the sampling effort in the case of a large ecosystem.

### Comparison of eDNA metabarcoding with complete electrofishing within the river

On the river, the low depth and width on sampling sites R1 and R2 allowed performing two complete electrofishing multi-passes removals in order to get a reliable absolute estimation of the fish community composition within the spatial limits of each of the two stations as well as temporal, in correlation to eDNA metabarcoding samplings. This allowed testing eDNA metabarcoding as a method for assessing the absolute local species richness.

On the river sampling sites R1 and R2, all the taxa sampled with the traditional survey are also detected by eDNA metabarcoding and this last technique is able to detect six and five additional species on R1 and R2, respectively. All these additional taxa were previously caught in the past along the Tier River and are ecologically relevant at the scale of the whole river. Considering that the three pass removal sampling method gives an estimate close to the absolute species richness at the sampling site, the higher number of species detected by eDNA metabarcoding demonstrated that this last technique describes the species diversity at a larger scale, including for river reaches located upstream.

### Longitudinal pattern of fish biodiversity and distance of detection

The spatial pattern of fish community structure described by all eDNA metabarcoding detections at the catchment scale allows a better characterization of the spatial representativeness of this method. The two river sampling stations (R1, R2) are clearly distinguishable from the lake samples on the CA factorial map, and also from one another. The species detections from the impoundment samples are quite comparable to the lake. Moreover, no typical fish lake species is detected two kilometers downstream from the dam and all eDNA metabarcoding samples are closely clustered per sampling site, with the exception of one R1 sample close to R2 samples. On the opposite, the eDNA R0 samples are characterized by a high variability due to the detection of lake dwelling species [40,45] in two samples, whereas these species are absent in the third sample (in particular *C*. *lavaretus*, *Salvelinus spp*., but also *C*. *Carpio*, *T*. *tinca*). The eDNA signals from typical lake species disappear quickly in the river, along the first kilometer downstream from the lake. In addition, the location and dispersion of the traditional samples from both the lake and the river fit well with those observed for eDNA samples, which demonstrates the capacity of eDNA metabarcoding to describe the succession of the different fish communities in space.

In our study, the distance of eDNA detection is around two to three kilometers, i. e. the distances between our sites. This value is intermediate between the ten kilometers found by Deiner and Altermatt [24] on lake dwelling species in natural ecosystems and the five meters estimated in the experiment of Pilliod *et al*. [23] based on individuals introduced in high density. The differences in water velocity between studied rivers could explain the longer distance of detection found by Deiner and Altermatt [24]. After release in water, eDNA remains detectable from a few days to a few weeks in mesocosms [19–22]. In our study area, the water coming from the lake takes approximately two days to reach the sampling site situated the furthest downstream (R2). Therefore, it seems that the distance of detection of the eDNA signal is less than its potential persistence, even if additional research is needed on different types of rivers including unimpounded rivers in terms of hydrological and physico-chemical conditions. Indeed, the state (intra or extracellular eDNA, aggregate size), localization (adsorption, biological uptake and other interactions between DNA molecules and the aquatic environment) and fate (biotic and abiotic conditions influencing the degradation rate) of aquatic macroorganism’s eDNA is poorly known and probably highly variable [44]. No eDNA sampling was performed downstream from the location where the impounded water flows back into the river, so it is not possible to know if lake species can be detected in this section of the river. Nevertheless, the range of detection distances with our method is adapted to describe fish biodiversity at the scale of a water body, i.e. the basic unit that is used to assess water quality in Europe and to set targets for environmental improvements [28]. In particular, the eDNA metabarcoding workflow developed by Valentini *et al*. [13] is of first interest from both the scientific and the socio-economic point of view when it comes to bypass the spatial limits of traditional sampling and to deliver a list of species globally equivalent to the cumulative effort of more than ten traditional sampling campaigns. Moreover, this approach is non-invasive and cost-effective as it needs much less equipment and less human resources per sampling site.

Our results also illustrate the need for more work on the definition of the sampling effort, in particular within very large ecosystems such as deep alpine lakes. The sampling strategy also has to be better investigated: types of samples (stationary or integrated), water volume per sample, effect of environmental conditions on sampling efficiency (e.g. hydrology, physicochemical characteristics, suspended mater, depth in lakes, etc.) and sample variability between habitats within the same sampling site. Currently, eDNA metabarcoding could be used to assess the whole biodiversity of a water body from one sample, but only in terms of presence/absence of species. Ecological assessments requiring estimations of population biomass or abundance still need further improvements of the eDNA metabarcoding method [46]. Undoubtedly, this question will be one of the main targets in this research field in the next years.
